# Fish consumption and cardiovascular response during mental stress

**DOI:** 10.1186/1756-0500-5-288

**Published:** 2012-06-13

**Authors:** Kenta Matsumura, Takehiro Yamakoshi, Hiroko Noguchi, Peter Rolfe, Yutaka Matsuoka

**Affiliations:** 1 Department of Adult Mental Health, National Institute of Mental Health, National Center of Neurology and Psychiatry, 4-1-1 Ogawa-Higashi, Kodaira, Tokyo, 187-8553, Japan; 2 Graduate School of Natural Science and Technology, Kanazawa University, Kakuma-machi, Kanazawa, Ishikawa, 920-1192, Japan; 3 Clinical Psychology Center, Musashino University, 3-40-10 Sekimae, Musashino, Tokyo, 180-0023, Japan; 4 Department of Automatic Measurement and Control, Harbin Institute of Technology, No.92 West Da-Zhi Street, Harbin, Heilongjiang, 150001, China; 5 Department of Psychiatry and Clinical Research Institute, National Disaster Medical Center, 3256 Midori-cho, Tachikawa, Tokyo, 190-0014, Japan; 6 CREST, Japan Science and Technology Agency, 3256 Midori-cho, Tachikawa, Tokyo, 190-0014, Japan; 7 Translational Medical Center, National Center of Neurology and Psychiatry, 4-1-1 Ogawa-Higashi, Kodaira, Tokyo, 187-8551, Japan

**Keywords:** Fish consumption, Hemodynamics, Impedance cardiography, Arterial stiffness, Coronary heart diseases, Omega-3 fatty acids

## Abstract

**Background:**

Frequent fish consumption is related to a lower risk of coronary heart disease. However, the physiological mechanisms underlying this cardioprotective effect are as yet unknown. We therefore examined certain cardiovascular physiological variables of fish eaters during rest, whilst conducting mental arithmetic, and during recovery.

**Findings:**

The participants were 12 fish eaters (eating baked fish more than 3–4 times/week) and 13 controls (eating fish less than 1–2 times/week). Analysis of the collected data revealed that heart rate, blood pressure, and pulse wave velocity were significantly lower and pre-ejection period and baroreflex sensitivity were significantly higher in the fish eaters than in the controls during both rest and mental arithmetic, and that systolic and mean blood pressure recovery from mental arithmetic were faster in the fish eaters than in the controls.

**Conclusions:**

These findings suggest a possible physiological mechanism that may explain why frequent fish consumption reduces coronary heart disease risk.

## Findings

### Introduction

Accumulating evidence from human meta-analysis and large-scale epidemiological studies has shown that frequent fish consumption protects against coronary heart disease (CHD) [1-4]. Recent studies on cardiovascular function and hemodynamics have attempted to elucidate the possible mechanisms underlying this cardioprotective effect. For example, frequent fish consumption in the elderly is associated with lower blood pressure, heart rate, and total peripheral resistance [5]. However, little is known about the effect of frequent fish consumption on cardiovascular behavior during acute mental stress. This is somewhat surprising considering that exaggerated cardiovascular reactivity to [6-9], and/or slow recovery from acute laboratory mental stress [10], that show striking individual differences from one’s early years [11,12], have been implicated in the pathogenesis of CHD.

As for the relationship between fish consumption and stress reactivity, to our knowledge, there are four related human studies [13-16]. These are studies that examined the effect of the supplementation of the omega-3 fatty acid such as docosahexaenoic acid (DHA) and eicosapentaenoic acid (EPA) on cardiovascular reactivity during stress. DHA and EPA is known to be almost exclusively contained in fish oil, and considered to be the main substance that protects against coronary heart disease [17]. Among these four studies, two [15,16] were intact randomized control trials (RCT), but the result was contrary; that is, one [15] was positive, the other [16] was negative. Although there were many differences between these two studies such as the stress task used and the dose of omega-3 fatty acids, one highly plausible explanation as to this discrepancy is that the duration of supplementation was too short to consistently affect stress reactivity and/or recovery. According to a meta-analysis study that sums up 30 RCTs [18], at least twelve weeks of supplementation is necessary to stably lower resting heart rate, but the duration of the above mentioned contradictory RCTs were 3 weeks and 4 weeks, respectively.

In the present study, therefore, we examined cardiovascular behavior of young fish-eaters at rest, during acute mental stress, and during recovery. Fish-eaters would have a diet that contains a high concentration of omega-3 fatty acid long enough to affect cardiovascular measures. To carry out this study, we recruited one group of individuals who had the habit of regularly eating fish and a second group who did not. The two groups then performed a laboratory mental stress task. Chosen cardiovascular indices were measured before, during, and after these mental stress tasks.

### Methods

#### Participants

We firstly established the criterion with which to discriminate between fish-eaters and non-fish eaters. Staple Japanese baked fish preparations usually exceed 70 g, which is significantly greater than the amount of other preparations of fish, such as raw fish, the amount of which is variable and relatively small. We therefore adopted the following simple operational classification to emphasize group differences.

##### Fish-eating group

A total of 12 fish eaters, defined as those who consumed more than 70 g of baked fish at least 3–4 times/week for at least the last year, participated in the study.

##### Control group

A total of 13 controls, which consumed less than 70 g of baked fish 1–2 times/week for at least the last year, participated in the study.

The participants were young students, responding to fliers or e-mails distributed by the experimenters. The criteria for inclusion in the study were to be 18 to 30 years of age, having no history of or current cardiovascular disease, and not taking any prescription medication. The participation of women was at random with regard to the menstrual cycle phase.

All the participants received 2,000 Yen (about US$ 25) as a reward for their cooperation. After providing a complete description of the study, written informed consent was obtained from all participants. This study was approved by the ethics committee of the National Disaster Medical Center, Tokyo.

#### Apparatus

The experiment was conducted in a 3 × 3-m sound-attenuated, temperature-controlled experimental room. A wooden desk and a comfortable chair for participants to sit on were made available in this room. Participants were monitored using a VGA resolution web camera and a condenser microphone (Rhodes, NT2A) placed on the desk. The stress task, described below, was controlled by a computer (Apple, MacOS X 10.4) situated in the control room, and was presented through a LCD monitor (BenQ, FP91G) and small speakers on the desk.

Beat-to-beat blood pressure (BP) was measured noninvasively using a finger-cuff attached to the middle finger of the left hand and connected to a BP monitor (Medisens, MUB101), utilizing the volume-compensation principle [19,20]. The measurements obtained using this device were found to be in good agreement with those obtained using a standard oscillometric monitor (GE Healthcare, Dinamap ProCare 100). The electrocardiogram (ECG) was recorded from a pair of disposable electrodes, attaching one electrode to each arm of the participants (that is, standard limb electrocardiogram, lead I), and connecting the electrodes to a bioamplifier (Monte system, ECG100C). The thoracic impedance cardiograms were obtained using 4 circumferential electrodes, which were placed around the participants’ neck and chest and connected to an impedance cardiograph (Monte system, NICO100C). The finger photo-plethysmogram was recorded using a near-infrared light emitting diode as a light source and a photo diode as a detector, which were placed on the index finger of the left hand and connected to a bioamplifier (Monte system, BIOPAC UIM100C). These signals were amplified using a bioamplifier (Monte system, BIOPAC MP150), sampled at a rate of 1000 Hz with a resolution of 16 bits and stored digitally in a computer (Apple, iMac).

#### Laboratory mental stress task

##### Baseline (BS) and recovery (R)

In both periods, the participants were asked to sit still and observe the blank computer screen.

##### Mental arithmetic (MA) task

The participants were instructed to subtract 13 sequentially from 5000 (to get 4987, 4974, 4961,…) as quickly and accurately as possible [21]. The participants were given voice feedback of “Yes” and “No” for every correct and incorrect answer, respectively. In the latter case, subtraction was continued from the correct answer. The scores achieved by the participants were displayed on the screen, with 2 points added for every correct answer and 1 point subtracted for every incorrect answer. The screen also displayed the target score, which was updated every 20 s, to encourage optimal performance. This score was determined according to the participant’s answering speed; that is, the first 20 s target score was 6 and below. If the participant attained this score, the next target score was 8 added to the present achieved score, but if not, the next target score was 4 added to the present achieved score. The minimum added score was 2, and the same rule was applied until the end of this task. An aversive, glass-scratching noise of 68 dB(A) was sounded for 1.5 s every 20 s if the response was delayed.

#### Procedure

The participants were asked in advance to maintain their regular lifestyle, to refrain from any medication before the study, and to avoid consumption of food and caffeine-containing substance, intense physical activity, and smoking for an hour before laboratory testing. After being fitted with instruments for cardiovascular monitoring, they sat on the chair placed before a 17-inch computer screen in the experimental room during the experiment. A 10-min rest period, with the last 3 min used as a baseline, was followed by the 5-min mental arithmetic task. Then, a 2-min post-task checklist period was followed by a 9-min recovery period.

#### Questionnaires

##### Food intake

The participants completed a Food Frequency Questionnaire (FFQ) that has been standardized as a dietary assessment tool for Japanese [22,23]. The validity and reliability of this FFQ has been confirmed in terms of accordance with the dietary record and blood sample, and 1-year interval reproducibility [24,25].

##### Psychometrics

The participants completed a Japanese standardized and validated version of the Buss-Perry Aggression Questionnaire (BAQ; [26]), the State-Trait Anxiety Inventory (STAI; [27]), the Short-Form Eysenck Personality Questionnaire-Revised (EPQR-12; [28]), and the Behavioral Inhibition/Activation (Approach) System Scale (BIS/BAS; [29]).

##### Post-task checklist

After they had finished the task, the participants answered 5 simple questions as to their subjective state during the task; attention to the display, unpleasant feelings, effort to cope, task difficulty, and perceived control. All items selected are known to affect cardiovascular reactivity [30-33]. Each item is written in a Likert-type format, with the response made on an 11-point response scale, with 0 indicating *not at all* and 10 indicating *extremely high*.

#### Cardiovascular measures and data reduction

Systolic, mean, and diastolic blood pressure (SBP, MBP, and DBP, respectively) were determined on a beat-by-beat basis. Heart rate (HR) was derived from the R-R intervals of the ECG. Cardiac output (CO) and pre-ejection period (PEP) were determined with a 60-s ensemble-averaging technique [34,35] using the ECG and the impedance cardiogram. Total peripheral resistance (TPR) was calculated as TPR = MBP/CO. Normalized pulse volume (NPV), an index of α-adrenalin-mediated sympathetic activity [36], was calculated by dividing the AC component of the finger photo-plethysmogram by its DC component and then applying a logarithmic transformation to normalize the distribution. Pulse-wave velocity (PWV), an index of arterial stiffness, was calculated as PWV = *D*/PTT, where *D* is the distance between the heart and finger, measured along the body surface, and PTT is the pulse transmission time, calculated as the interval between the pulse wave reaching the finger (DBP point of the BP wave) and the ejection of the blood from the left ventricle (*B* point of the d*Z*/d*t* wave). The cardio-finger vascular index (CFVI), which represents arterial stiffness independent of the BP [37], was calculated as CFVI = [1/(SBP - DBP)] × *ln*(SBP/DBP) × PWV^2^. The baroreflex sensitivity (BRS), an index of cardiac vagal activity, was computed using the sequence method [38].

Beat-by-beat SBP, MBP, DBP, HR, and *ln* NPV values were averaged over 60 s, and PEP, CO, TPR, PWV, CFVI, and BRS were calculated for the same duration. Subsequently, these values were further averaged to produce BS, MA, and first (0–3 min), second (3–6 min), and third (6–9 min) recovery (R1, R2, and R3, respectively) values, respectively. Reactivity and recovery values were calculated by subtracting BS values from MA values and the mean of these R values; that is, R1, R2, and R3, respectively.

#### Statistical analyses

Physiological data for each group (fish-eating and control) and condition (BS, MA, R1, R2, and R3) were compared statistically by means of a series of separate two-way mixed-design analyses of variance (ANOVAs). The Greenhouse-Geisser correction was applied to the degree of freedom where appropriate. Tukey’s Honestly Significant Difference tests (Tukey *HSD*) for post-hoc comparison were used with a significance level of 5%. Unpaired *t*-tests between groups were also conducted both in reactivity and recovery values. In the case of problems in the measurement of a certain index across a condition, all the measurements of the index in the particular participant were treated as missing data. Other group differences such as diet and psychometrics were compared statistically using unpaired *t*-tests or Chi-square tests with Yates’ continuity correction. All analyses were performed using IBM SPSS Statistics 18.0 for MacOS (IBM Inc.).

### Results

#### Characteristics of the fish-eating and control groups

The descriptive characteristics of the fish-eating and control groups are summarized in Table 1. The results of statistical tests are also presented in Table 1.

**Table 1:** Demographic, anthropometric, dietary, and psychometric characteristics of participants with different fish-eating habits

**Characteristics**	**Group**		***Statistic***	***p***	***ES***
	**Fish-eating**	**Control**			
	***M* (*SD*)**	***M* (*SD*)**			
Demographics			*χ*_1_^2^		*φ*
Gender (M/F)	2/10	2/11	0.00	n.s.	0.00
Smokers (Y/N)	0/12	1/12	0.00	n.s.	0.00
Anthropometrics			*t*_23_		*d*
Age (years)	21.4 (3.7)	21.9 (3.1)	0.37	n.s.	0.15
Body Mass Index (kg/m^2^)	21.6 (2.7)	20.8 (3.1)	0.67	n.s.	0.28
Food Intakes (FFQ) (g/day)					
Eating Fish (times/week)	3.8 (0.8)	1.0 (0.6)	9.92	<.001	4.14
Fish and Seafood	91.7 (34.7)	48.2 (32.7)	3.22	<.004	1.34
Mushroom	5.4 (4.7)	5.9 (5.4)	0.25	n.s.	0.10
Algae	7.5 (3.7)	3.9 (3.7)	2.40	<.03	1.00
Alcohol	39.1 (101.1)	60.8 (195.7)	1.09 ^a^	n.s.	0.44
Fruits	111.9 (88.9)	44.4 (52.7)	2.33	<.03	0.97
Vegetables	367.8 (251.0)	230.7 (120.7)	1.72 ^b^	<.10	0.74
Green and Yellow Vegetables	144.6 (84.0)	93.8 (70.2)	1.65	n.s.	0.69
Cereal	256.2 (88.9)	203.3 (92.7)	1.45	n.s.	0.61
Processed Tubers and Roots	57.8 (58.1)	50.6 (48.8)	0.34	n.s.	0.14
Pulse Products	57.9 (49.6)	56.3 (59.5)	0.07	n.s.	0.03
Meat	107.8 (60.2)	103.0 (71.2)	0.18	n.s.	0.08
Egg	48.7 (34.4)	29.1 (17.5)	1.81	<.09	0.75
Dairy Product	91.7 (152.1)	146.5 (132.7)	0.96	n.s.	0.40
Psychometrics					
Aggression (BAQ)	56.3 (7.3)	50.9 (10.0)	1.00	n.s.	0.63
Trait Anxiety (STAI A-trait)	35.2 (12.1)	40.5 (9.3)	1.23	n.s.	0.51
Neuroticism (EPQR-12)	5.0 (2.8)	7.2 (2.4)	2.07	<.06	0.86
Behavioral Inhibition (BIS)	39.0 (6.4)	38.8 (10.0)	0.07	n.s.	0.03
Behavioral Activation (BAS)	45.0 (8.7)	38.6 (5.6)	2.20	<.04	0.92

*Note*. *χ*^2^ = Yates corrected Chi-square.

^a^*df* = 14; ^b^*df* = 15.

#### Post-task checklist

Subjective ratings for the MA task are summarized in Table 2. The results of statistical tests are also presented in Table 2.

**Table 2:** Subjective rating to the mental arithmetic task

**Post-Task Checklist**		**Group**		***t*_23_**	***p***	***d***
		**Fish-eating**	**Control**			
		***M* (*SD*)**	***M* (*SD*)**			
Items						
	Attention to the Display	3.8 (1.7)	5.5 (3.3)	1.73 ^a^	n.s.	0.70
	Unpleasant Feelings	6.5 (2.7)	6.7 (2.3)	0.19	n.s.	0.08
	Effort to Cope	7.8 (1.9)	8.3 (1.3)	0.74	n.s.	0.31
	Task Difficulty	8.0 (1.7)	8.7 (1.8)	0.99	n.s.	0.41
	Perceived Control	3.8 (1.9)	3.2 (2.5)	0.76	n.s.	0.32

*Note*. 11-point rating from 0 (not at all) to 10 (extremely high).

^a^*df* = 17.

#### Cardiovascular measures

**Figure 1:**
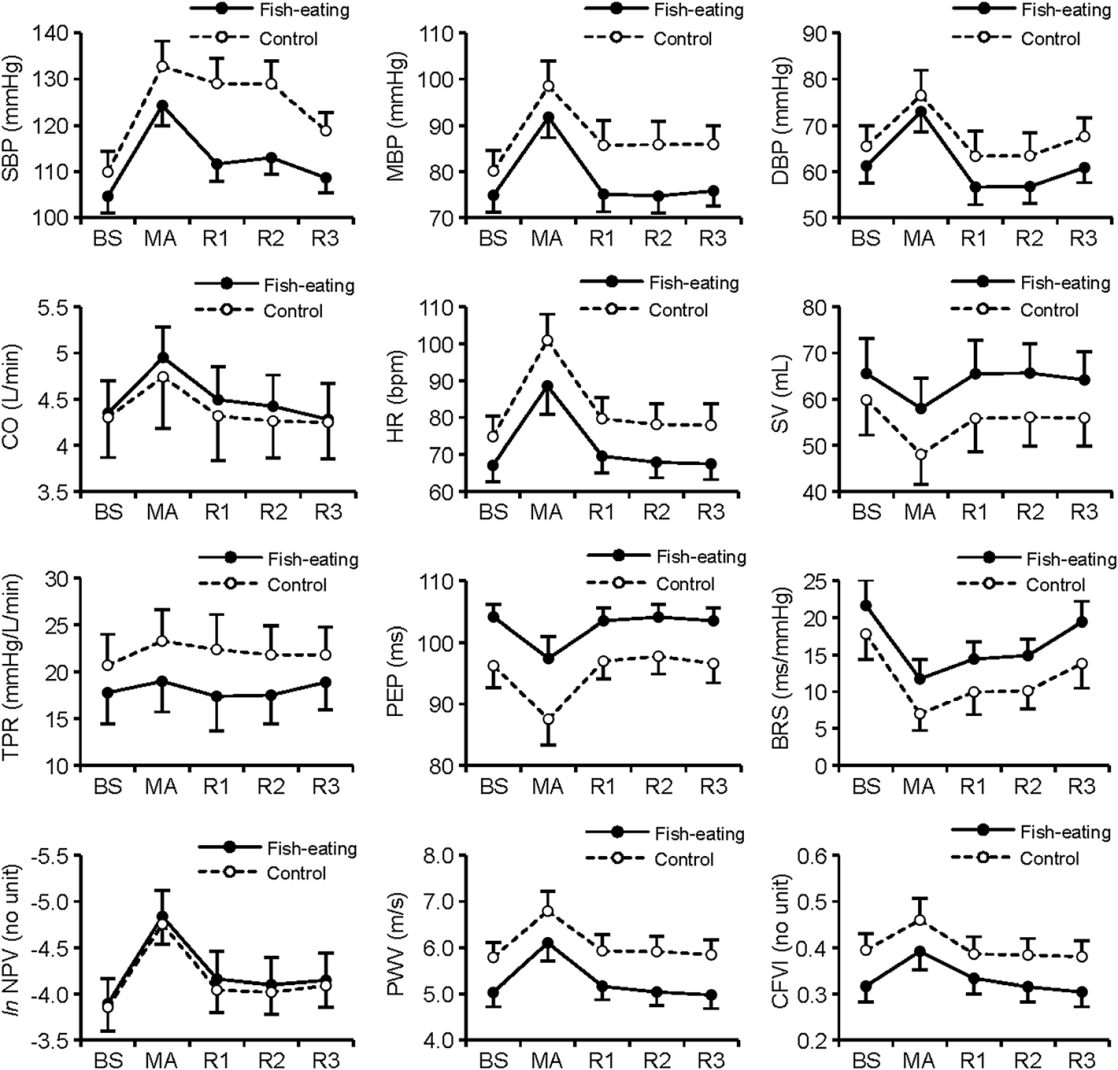
**Cardiovascular responses during baseline, mental stress, and recovery.** Means (*S.E.M.*) of systolic, mean, and diastolic blood pressure (SBP, MBP, and DBP, respectively), cardiac output (CO), heart rate (HR), stroke volume (SV), total peripheral resistance (TPR), pre-ejection period (PEP), baroreflex sensitivity (BRS), *ln* normalized pulse volume (*ln* NPV), pulse wave velocity (PWV), and cardio-finger vascular index (CFVI) during baseline (BS), mental arithmetic (MA), and the 1^st^, 2^nd^, and 3^rd^ recovery (R1, R2, and R3, respectively).

Cardiovascular responses are shown in Figure 1. The results of a series of Group (fish-eating and control) × Condition (BS, MA, R1, R2, and R3) ANOVAs and of their post-hoc tests are summarized in Table 3. We also conducted ANOVAs using BS values as covariate, but results hardly changed. Therefore, we only represent the results of simple ANOVAs.

**Table 3:** Summary of a series of separate Analyses of Variances (ANOVAs)

**Cardiovascular Measures**			**Main Effect**							**Interaction**				**Summary of ANOVA and Post-hoc Test**
			**Group**			**Period**				**Group × Period**				
			***F*_1, 23_**	***p***	***η*_*p*_^2^**	***F*_4, 92_**	***p***	***ϵ***	***η*_*p*_^2^**	***F*_4, 92_**	***p***	***ϵ***	***η*_*p*_^2^**	
Hemodynamics														
		SBP	6.15	<.03	.21	30.84	<.001	.79	.57	3.10	<0.05	.79	.12	FE < Cont on R1, R2, and R3
		MBP	5.47	<.03	.19	56.56	<.001	.76	.71	1.94	n.s.	.76	.08	FE < Cont; BS, R2, R1, R3 < MA
		DBP	4.04	<.06	.15	78.19	<.001	.62	.77	1.37	n.s.	.62	.06	FE < Cont; R1, R2 < BS, R3 < MA
	Cardiac													
		CO	0.08	n.s.	.00	5.84	<.006	.49	.20	0.17	n.s.	.49	.01	R3, BS, R2, R1 < MA
		HR	5.55	<.03	.19	65.52	<.001	.32	.74	0.47	n.s.	.32	.02	FE < Cont; BS, R3, R2, R1 < MA
		SV	1.68	n.s.	.07	10.66	<.001	.70	.32	0.58	n.s.	.70	.02	BS, R2, R1, R3 > MA
	Vascular													
		TPR	2.52	n.s.	.10	2.28	n.s.	.74	.09	0.90	n.s.	.74	.04	
Autonomic Activities														
	Sympathetic													
		PEP	10.32	<.004	.31	25.75	<.001	.42	.53	1.10	n.s.	.42	.05	FE > Cont; R2, R1, BS, R3 > MA
		*ln* NPV	0.10 ^a^	n.s.	.00	46.5 ^b^	<.001	.68	.68	0.08 ^b^	n.s.	.68	.00	BS, R2 > R2, R1, R3 > MA
	Vagal													
		BRS	4.39 ^c^	<.05	.17	26.29 ^d^	<.001	.63	.56	0.16 ^d^	n.s.	.63	.01	FE > Cont; BS, R3 > R2, R1 > R1, MA
Arterial Stiffness														
		PWV	10.33	<.004	.31	73.73	<.001	.34	.76	0.56	n.s.	.34	.02	FE < Cont; BS, R3, R2, R1 < MA
		CFVI	6.09	<.03	.21	28.27	<.001	.47	.55	0.61	n.s.	.47	.03	FE < Cont; R3, R2, BS, R1 < MA

*Note*. ^a^*df* = 1, 22; ^b^*df* = 4, 88; ^c^*df* = 1, 21; ^d^*df* = 4, 84.

Period(s) separated by comma and printed both sides of inequality sign are not significantly different at *p* < .05.

*FE* = fish-eating, *Cont* = control.

*BS* = baseline, *MA* = mental arithmetic, *R1* = 1^st^ recovery, *R2* = 2^nd^ recovery, and *R3* = 3^rd^ recovery.

*SBP* = systolic blood pressure, *MBP* = mean blood pressure, *DBP* = diastolic blood pressure, *CO* = cardiac output, *HR* = heart rate, *SV* = stroke volume, *TPR* = total peripheral resistance, *PEP* = pre-ejection period, *NPV* = normalized pulse volume, *BRS* = baroreflex sensitivity, *PWV* = pulse wave velocity, and *CFVI* = cardio-finger vascular index.

The reactivity and recovery values together with the results of unpaired *t*-tests are presented in Table 4.

**Table 4:** Cardiovascular reactivity and recovery values

**Cardiovascular Measures**			**Reactivity**					**Recovery**				
			**Group**		***t*_23_**	***p***	***d***	**Group**		***t*_23_**	***p***	***d***
			**Fish-eating**	**Control**				**Fish-eating**	**Control**			
			***M* (*SD*)**	***M* (*SD*)**				***M* (*SD*)**	***M* (*SD*)**			
Hemodynamics												
		SBP, mmHg	19.7 (7.7)	22.8 (12.2)	0.77	n.s.	0.31	6.5 (7.8)	15.7 (11.5)	2.32	<.03	0.95
		MBP, mmHg	16.9 (5.8)	18.4 (8.6)	0.49	n.s.	0.20	0.4 (4.4)	5.7 (5.9)	2.53	<.02	1.02
		DBP, mmHg	11.7 (4.5)	11.0 (4.7)	0.40	n.s.	0.16	−3.2 (4.1)	−0.7 (5.0)	1.36	n.s.	0.55
	Cardiac											
		CO, L/min	0.60 (0.66)	0.44 (1.20)	0.42	n.s.	0.17	0.05 (0.46)	−0.03 (0.41)	0.44	n.s.	0.18
		HR, bpm	21.4 (15.1)	26.1 (12.1)	0.87	n.s.	0.35	1.3 (3.1)	3.8 (5.1)	1.47	n.s.	0.61
		SV, mL	−7.6 (8.5)	−11.7 (13.0)	0.94 ^a^	n.s.	0.38	−0.5 (7.3)	−3.9 (6.3)	1.25	n.s.	0.50
	Vascular											
		TPR, mmHg/L/min	1.2 (2.5)	2.6 (4.4)	0.93	n.s.	0.39	0.2 (2.2)	1.3 (2.1)	1.33	n.s.	0.53
Autonomic Activities												
	Sympathetic											
		PEP, ms	−6.7 (6.7)	−8.7 (7.9)	0.66	n.s.	0.27	−0.4 (2.0)	0.9 (4.4)	0.97	n.s.	0.41
		*ln* NPV	−0.94 (0.31)	−0.90 (0.54)	0.24 ^b^	n.s.	0.10	−0.24 (0.36)	−0.20 (0.30)	0.32 ^b^	n.s.	0.13
	Vagal											
		BRS, ms/mmHg	−9.9 (8.4)	−10.8 (7.1)	0.28 ^c^	n.s.	0.12	−5.4 (5.8)	−6.5 (4.0)	0.53 ^c^	n.s.	0.23
Arterial Stiffness												
		PWV, m/s	1.06 (0.57)	1.00 (0.46)	0.30	n.s.	0.12	0.03 (0.17)	0.11 (0.20)	1.08	n.s.	0.44
		CFVI	0.08 (0.07)	0.07 (0.06)	0.39	n.s.	0.16	0.00 (0.03)	−0.01 (0.04)	0.94	n.s.	0.38

*Note*. ^a^*df* = 20; ^b^*df* = 22; ^c^*df* = 21.

*SBP* = systolic blood pressure, *MBP* = mean blood pressure, *DBP* = diastolic blood pressure, *CO* = cardiac output, *HR* = heart rate, *SV* = stroke volume, *TPR* = total peripheral resistance, *PEP* = pre-ejection period, *NPV* = normalized pulse volume, *BRS* = baroreflex sensitivity, *PWV* = pulse wave velocity, and *CFVI* = cardio-finger vascular index.

### Discussion

In this laboratory study, the fish-eating group showed distinct and unique cardiovascular response patterns before, during, and in recovery from MA, as compared to the control group. Specifically, lower BP, HR, PWV, and CFVI, higher BRS and PEP, and faster SBP and MBP recovery were observed in the fish-eating group. Such patterns clearly represent healthy cardiovascular responses and arterial elasticity of the fish-eating group, and this is highly consistent with that observed in previous studies. For example, as mentioned above, one study [5] that examined elderly people revealed that fish-eaters had lower HR and BP. Although the results in our study did not reach a statistically significant level, their study detected lower TPR and higher SV, and comparable CO. Similarly, our finding of lower PWV and CFVI, reflecting arterial stiffness independent of BP, corresponds well with a study [39] that examined atherosclerosis of middle-aged Japanese, Japanese-American, and White men in relation to fish consumption. Frequent fish consumption is highly likely to promote cardiovascular health in a younger population, as it appears to in the older populations.

Faster SBP and MBP recovery following MA was observed in the fish-eating group. On the other hand, no reduced reactivity to MA was detected in any indices, even though some raw values during MA in the fish-eating group were different from those in the control group. As clearly seen in Figure 1, the slopes of the lines through points BS and MA in two groups were the same, thus group differences in reactivity, defined as changes from baseline to the task [40], were not observed. In relation to these unique response patterns, some researchers have proposed an interesting view that what is causally related to atherosclerosis are higher or lower raw values in themselves, rather than higher reactivity to the stress [41,42]. As an example, 70 bpm HR during baseline increased to 100 bpm during stress and 80 bpm HR during baseline increased to 110 bpm during stress are comparable in terms of reactivity, but the latter would be worse for future cardiovascular health. According to this view, it follows that the effect of frequent fish consumption on cardiovascular health should be mediated by modulations in raw values, rather than reduced reactivity. Further studies on this aspect would be needed.

We measured subjective ratings during MA using a post-task checklist, but no group differences were observed. Thus, these inner states do not noticeably affect cardiovascular measures in this study. However, these results do not necessarily rule out the possibility that relatively small sample size simply made it difficult to reach a statistically significant level. Thus, in future research, adopting a more detailed or standardized subjective ratings questionnaire such as Attention-Affect Check List (AACL; [43]), or manipulating these variables in a positive manner such as by using previously established paradigm [44], will be needed.

We also asked participants to answer psychometrics, yet the scores for the behavioral approach (activation) system (BAS) were higher in the fish-eating group. Although higher BAS score have already been shown to be related to higher HR reactivity [45], our cardiovascular data did not show such a tendency. This is possibly due to the characteristic feature of our MA task, in that there was no monetary bonus, but instead a glass-scratching noise punishment, and both groups of participants felt higher task difficulty and unpleasant feelings and lower perceived control, and as such, the BAS system was not activated.

A group difference in fish-eating habit was a salient feature, but such a dietary pattern was accompanied by modulation in other food categories, including a higher intake amount of fruits, algae, and vegetables. Besides algae, that was only recorded at very low intakes and is similar to fish, being marine-derived, fruits and vegetables are known to be good for cardiovascular health [46], thus, in theory, their possible effects in our present study can not be ignored. However, at present, the relationship between consumption of fruits and vegetables and cardiovascular responses during mental stress is not established. In contrast, the cardiovascular response patterns observed in our fish-eating group were in quite good agreement not only with those observed in previous fish-eater studies, as mentioned above, but also with those obtained from studies supplementing fish oil to participants [47,48], and it is very difficult to explain such distinct and unique patterns from the viewpoint of other factors. Thus, at least as with respect to cardiovascular measures, it is likely that the group differences were mainly due to the difference of fish-eating habit.

In relation to the above, fish-eating is considered to be a healthy diet in Western countries. In this study, we simply asked the participants after finishing the experiment why they ate fish. Their answers were either that “I like fish” or “it is prepared in my house”, or both of these. These answers clearly show the attitude of Japanese to eating fish; that is, the majority of Japanese do not regard fish as a healthy diet, but as a delicious and/or traditional one. Thus, at least with regard to our participants, it is very unlikely that a high health attitude promotes other healthy behaviors, such as exercise, which in turn affect cardiovascular measures.

There are some limitations in this study. Firstly, although the differences seen in our cardiovascular data appeared to be statistically significant, the relatively small sample size makes the use of covariate analysis to adjust for possible confounding factors, such as personality traits and dietary habits other than fish, rather difficult. Thus, future studies, in which larger sample sizes are recruited, are needed, though young people are rarely expected to be classified into a fish-eating group. Secondly, we only used the MA task, but there are other tasks that evoke different hemodynamic reaction patterns. It could be fruitful to use a cold pressor test, which can provoke vascular responses, or a real-life competitive situation as an intense stressor [49]. Thirdly, we did not measure cardiovascular indices during the 2-min checklist period. Although we have assumed that the measured variables would gradually recover from the task, we cannot exclude the possibility that a relevant acute event might have occurred. So, in future studies, procedures that enable us to record both physiological and psychological measures simultaneously should be adopted. Despite these limitations, our study revealed that fish-eaters show a more favorable cardiovascular response to a mental stress task and better arterial elasticity than non-fish eaters. Taken together with the slowly developing nature of cardiovascular disease, the long succession of such psychophysiological modulation might suggest a potential underlying mechanism by which frequent fish consumption protects against CHD.

## Abbreviations

CHD, Coronary heart disease; DHA, Docosahexaenoic acid; EPA, Eicosapentaenoic acid; RCT, Randomized control trial; FFQ, Food Frequency Questionnaire; BAQ, Buss-Perry Aggression Questionnaire; STAI, State-Trait Anxiety Inventory; EPQR-12, Short-Form Eysenck Personality Questionnaire-Revised; BIS, Behavioral Inhibition System; BAS, Behavioral Activation (Approach) System; BS, Baseline; MA, Mental arithmetic; R1, 1st recovery; R2, 2nd recovery; R3, 3rd recovery; SBP, Systolic blood pressure; MBP, Mean blood pressure; DBP, Diastolic blood pressure; CO, Cardiac output; HR, Heart rate; SV, Stroke volume; TPR, Total peripheral resistance; PEP, Pre-ejection period; BRS, Baroreflex sensitivity; NPV, Normalized pulse volume; PTT, Pulse transmission time; PWV, Pulse wave velocity; CFVI, Cardio-finger vascular index.

## Competing interests

The authors declare that they have no competing interests.

## Authors’ contributions

KM conceived and designed the study, performed the experiment and the analysis and drafted the manuscript. TY helped to conceive and design the study, performed the analysis, and helped to draft the manuscript. HN performed the experiment and the analysis. PR revised the manuscript critically. YM revised the manuscript critically and gave final approval of the version to be published. All authors read and approved the final manuscript.

## Authors’ information

Kenta Matsumura and Takehiro Yamakoshi are now at School of Mechanical Engineering, College of Science and Technology, Kanazawa University, Japan. Hiroko Noguchi is now at Translational Medical Center, National Center of Neurology and Psychiatry, Japan.
